# Optical Coherence Tomographic Finding in a Case of Congenital Macular Coloboma at King Abdulaziz University Hospital, Jeddah

**DOI:** 10.7759/cureus.14034

**Published:** 2021-03-22

**Authors:** Rahaf A Mandura, Rwan E Radi

**Affiliations:** 1 Department of Ophthalmology, King Abdulaziz University, Jeddah, SAU; 2 Department of Ophthalmology, College of Medicine, Umm Al-Qura University, Mecca, SAU

**Keywords:** macular coloboma, chorioretinal coloboma, optical coherence tomography, macular scars

## Abstract

Macular coloboma is a rare eye condition that affects around 0.5-0.7/10,000 of live births. Macular coloboma appears as a well-demarcated atrophic lesions that could affect one eye or both eyes on fundus examination. This is a case of a 33-year-old male patient who presented to the outpatient clinic with a history of poor vision in the left eye since childhood. He had a history of strabismus surgery for sensory exotropia (XT) in the left eye. Anterior segment examination of both eyes was normal while the fundus examination of both eyes revealed bilateral chorioretinal lesions in the macula which was larger in the left eye (OS) than the right eye (OD), representing bilateral chorioretinal coloboma. Congenital coloboma is a rare eye condition that leads to non-progressive decrease in visual acuity. Optical coherence tomography (OCT) is the modality of choice in diagnosing and describing macular coloboma.

## Introduction

Coloboma is an ocular congenital defect that can affect different areas of the eye such as the iris, the retina, the choroid, the lens or the optic nerve [1]. Meanwhile, macular coloboma is an atypical coloboma that is hypothesized to be the result of incomplete differentiation of the arcuate bundle along the horizontal raphe during development [2]. Macular coloboma is a rare eye condition that may affect about 0.5-0.7/10,000 live births [3]. On fundus examination, macular coloboma appears as well-demarcated atrophic lesions which can be unilaterally or in rare cases bilaterally [3]. Patients affected with macular coloboma suffer from non-progressive decrease in visual acuity [4]. Few cases of macular coloboma are associated with an inheritance pattern. The most common genetic inheritance is autosomal dominant while the rest of the cases are presented as an isolated cases with no known inheritance pattern [3]. Furthermore, the condition may be associated with various syndromes such as patau syndrome, Fraser syndrome, franceschetti syndrome, cat eye syndrome and many others [1]. Optical coherence tomography (OCT) is the most commonly used tool for the diagnosis of macular coloboma [4]. This is a case report of a case of bilateral macular coloboma with strabismus and sensory exotropia reported from king Abdulaziz University Hospital, Jeddah.

## Case presentation

**Figure 1:**
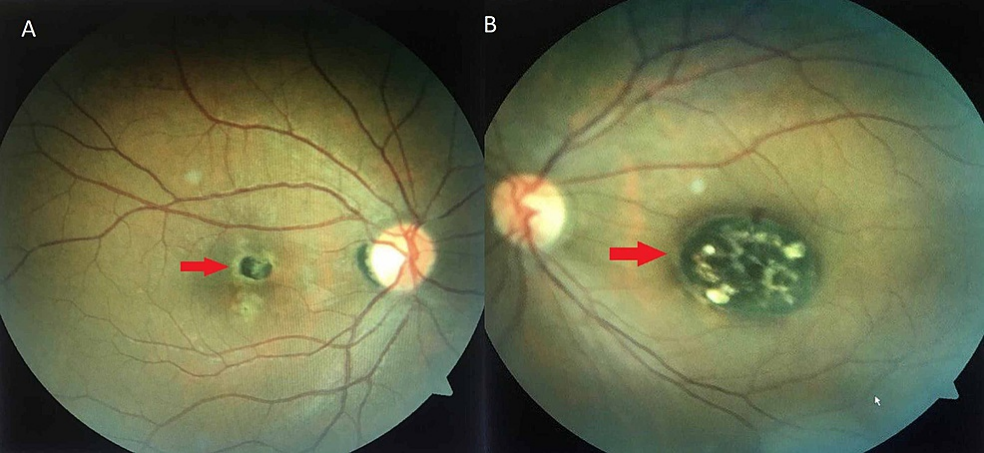
Macular coloboma: (A) right eye; (B) left eye.

**Figure 2:**
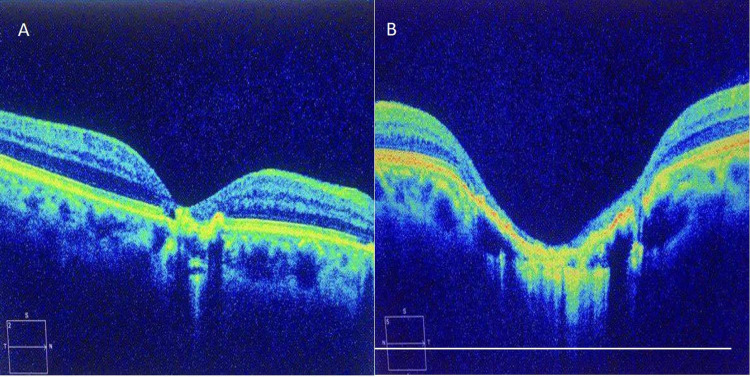
Optical coherence tomography: (A) right eye; (B) left eye.

A 33-year-old male patient presented to our outpatient clinic with a history of poor vision in the left eye since childhood. On eliciting the past ocular history, he had left eye strabismus surgery six years ago to correct sensory exotropia (XT). Previous medical history was positive for type 1 diabetes mellitus, and is controlled by insulin injections. His family history of ocular disease and systemic disease was unremarkable. Ophthalmic examination showed that the best-corrected visual acuity of the right eye (OD) was 20/30, and the left eye (OS) was hand motion. Intraocular pressure (IOP) on the non-contact tonometer was 16 mmHg and 12 mmHg in the right and left eye, respectively. The anterior segment examination was unremarkable in both eyes. Eye alignment by Hirschberg test and cover-uncover test showed orthotropic alignment in both eyes. Extraocular muscle movement showed a full range of movement without limitation bilaterally. The fundus examination (OD) showed a small circular well-circumscribed pigmented chorioretinal atrophic lesion. It had a well and sharp demarcated borders measuring around a half-disc diameter in both horizontal and vertical dimensions without involving the center of the fovea (Figure 1). Fundus examination (OS) revealed a large macular oval-shaped well-circumscribed excavated chorioretinal atrophic lesion centered in the fovea with visible large choroidal blood vessels against the white scleral background of the defect. The lesion was pigmented and surrounded by a dark, sharply demarcated rim of the retinal pigment epithelium (RPE) with some retinal vessels detected at the margin. It measured around two-disc diameter horizontally, and one-and-a-half-disc diameter vertically (Figure 1). Moreover, optic disc appearance on fundus exam was normal with no signs of diabetic retinopathy changes in both eyes. OCT model 2000 (Seuss-Humphrey Instrument Inc., San Leandro, CA, USA) was performed for both eyes and showed (OD) neurosensory retina is abnormally atrophied while the RPE showed preservation of the choroid (Figure 2). OCT (OS) showed a crater-like depression and an atrophic neurosensory retina with the absence of retinal pigment epithelium and choroid (Figure 2). Based on the clinical examination and OCT imaging, the patient was diagnosed as bilateral macular coloboma associated with left amblyopia with surgically corrected left sensory exotropia. In order to exclude congenital toxoplasmosis, which causes chorioretinal scarring in the macular area similar to the clinical appearance of macular coloboma, the patient was tested for anti-toxoplasmosis antibodies, which showed negative results.

## Discussion

Macular coloboma results from incomplete or poor differentiation of arcuate bundles along the horizontal raphe, which normally occurs during fetal development [4,5]. On the other hand, ocular toxoplasmosis leads to post-inflammatory macular scars that can mimic the presentation of macular coloboma [6]. Those scars are very similar in morphology to the scars of the congenital macular coloboma making it difficult to differentiate between congenital macular coloboma and post-inflammatory scars. The treatment course is usually determined based on the differentiation between different macular scars causes. OCT is the most commonly used tool for the diagnosis of macular coloboma [4,7].

Absence of the retina and choroid layers was reported using OCT in our patient. These findings are comparable to what has been reported in the literature [4,7-10]. Furthermore, many of macular coloboma cases are associated with other congenital syndromes and disorders [1]. However, our patient did not have any other congenital abnormality nor any family history of genetic diseases. Post-inflammatory macular scars due to congenital toxoplasmosis show different OCT findings that include thinning of the retina, cysts in the retina, fibrosis of the retina retinal pigment epithelium hyper-reflectivity which was in line with findings of our case and the diagnosis of bilateral congenital macular coloboma was established based on it [11,12]. Previous reports of patients with macular coloboma showed a visual acuity ranging from light perception to 20/20 [13-15]. In comparison, our patient had a poor visual acuity of hand motion in the left eye with large macular coloboma involving the center of the fovea, and good visual acuity of 20/30 in the right eye with a small coloboma that does not involve the center of the fovea.

Macular coloboma, especially those located at the posterior pole, can lead to retinal detachment leading to an increase risk of developing complete vision loss [3]. Therefore, regular follow-up for patients with macular coloboma is indicated. During each follow up a dilated fundus examination should be done for the patient in order to elicit any breaks at the coloboma edges [3]. Treatment of macular coloboma using laser photocoagulation is uncle limited in its role in preserving the vision. However, laser photocoagulation can be used for macular colobomas located away from the macula [3]. In addition, patients with macular colobomas should be treated carefully for any refractive errors to maximize and optimize the patient's visual acuity [3].

## Conclusions

Congenital coloboma is a rare ocular condition that leads to non-progressive decrease in visual acuity and if not followed up regularly may cause retinal detachment and vision loss. Optical coherence tomography is the method of choice in diagnosing macular coloboma while treatment should be done by laser photocoagulation if the coloboma is away from the macula with careful observation and follow-ups.
